# Supplementary material for: Are carbon emissions trading and green financial instruments synergistic? -Comprehensive quantitative research based on content analysis
Source: PLoS One. 2024 Mar 7;19(3):e0298601. doi: 10.1371/journal.pone.0298601 (PMC10919601; doi:10.1371/journal.pone.0298601)
Supplement: S1 Data — (ZIP) [file pone.0298601.s001.zip › Data/地级市Z_F工作报告.zip!/╜¡╬≈/╨┬╙α╩╨/└┤╫╘╣1⁄2╓┌║┼í╛┬φ┐╦╩2╛▌═°í┐_2011.docx]

各位代表：

　　现在，我代表市人民政府作政府工作报告，提请会同《新余市国民经济和社会发展第十二个五年规划纲要（草案）》一并审议，诚请各位市政协委员和列席会议的同志提出意见。

　　一、2010年及“十一五”时期主要情况回顾

　　2010年，面对极为复杂的经济社会发展环境和历史罕见的特大洪涝灾害，在省委、省政府和市委的正确领导下，全市上下坚持以科学发展观为指导，紧紧围绕“积极融入鄱阳湖生态经济区，加快建设国家新能源科技城”的经济工作主线和“通过10年努力，实现人均期望寿命80岁”的民生工程主线，团结拼搏，加压奋进，实现了经济社会又好又快发展，全面完成了市七届人大六次会议确定的各项目标任务，超额完成了“十一五”规划确定的主要经济指标。生产总值超出计划目标271.2亿元，财政总收入超出20.6亿元，地方财政收入超出20亿元，规模以上工业增加值超出70.4亿元，五年累计完成投资超出978.9亿元，外贸进出口总额超出26.1亿美元，社会消费品零售总额超出23亿元，城乡居民收入分别超出1358元、1276.2元。“十一五”时期主要成效集中反映为“八个明显”：

　　——综合实力明显增强。2010年全市生产总值631.2亿元，是“十五”末的3.6倍，年均增长15.5%。财政总收入80.6亿元，是“十五”末的3.5倍，年均增长28.5%。规模以上工业增加值270.4亿元，是“十五”末的4.4倍，年均增长34.5%；主营业务收入1230.3亿元，是“十五”末的5.2倍，年均增长39.1%。全社会固定资产投资658.3亿元，是“十五”末的7.4倍，年均增长50.8%。

　　——经济结构明显优化。三次产业结构由12.5:55.7:31.8调整为6:63:31，工业主导型经济增长格局进一步凸显。所有制结构日趋优化，非公经济增加值占生产总值比重由35.4%上升到62.2%。产品结构日趋优化，高新技术产品增加值占生产总值比重由6.95%提高到15%。荣获“中国城市科学发展转变经济发展方式示范城市”称号。

　　——城乡面貌明显改观。主城区面积由45平方公里扩大到64平方公里，城镇人口由33万增加到64万，城镇化率由48%提高到58.5%。新农村建设扎实推进，1472个新农村建设点投入建设资金11亿元。11个中心镇建设全面推进。率先在全省推行城乡公交一体化。

　　——生态环境明显改善。完成了万元生产总值能耗五年下降20%的目标。城市饮用水水质达标率为100%，仙女湖、孔目江两个饮用水源保护区内地表水质达Ⅱ类，袁惠渠、袁河地表水质达Ⅲ类。城区空气质量达国家二级标准。城镇生活污水集中处理率和生活垃圾无害化处理率分别达80%和90%。全市森林覆盖率达57.8%。被评为国家森林城市、国家园林城市和全省生态园林城市。

　　——创新能力明显提升。五年来，共建设国家级研发技术平台3个、国家级高新技术产业化基地2个，省级研发技术平台5个、市级工程技术中心14个，实施省重大高新产业化项目8项，新增省级创新型企业7家、省优势科技创新团队4个。全社会研发经费占生产总值比重由0.97%提高到2.6%，科技进步对经济增长的贡献率由45%提高到55%。国家知识产权示范城创建有序推进。被科技部授予全国唯一的“国家新能源科技示范城”称号。

　　—？？社会事业明显进步。基础教育工作居全省前列，义务教育均衡发展全面推进，在全省率先实施“幼儿进园、小学进镇、中学进城”工程。职业教育在校生由6.8万人增加到9.3万人，并率先在全省实行新余籍初、高中毕业生就读本市职业院校补贴政策，启动江西省职业教育园区建设。新余高专成功升本，填补了我市无本科院校的空白。市直三家公立医院均晋升为三级甲等医院，15所乡镇中心卫生院改造全面完成；率先在全省实现农村新型合作医疗和社区卫生服务机构全覆盖。文化广播影视体育事业取得新进步。我市连续五届获“全国双拥模范城市”称号。

　　——生活水平明显提高。城乡居民收入分别达17358元和7476.2元，年均分别增长13.9%和14.3%。社会消费品零售总额113亿元，年均增长19.4%。城镇人均住房建筑面积达32平方米，比“十五”末增加7平方米；农村人均居住面积达47平方米，比“十五”末增加17平方米。人均期望寿命由“十五”末的74.8岁提高到76.4岁。

　　——发展环境明显优化。致力打造与市场经济相适应的政务环境，先后荣获中国光电产业最佳投资城市、中国最具特色金融生态示范城市、中国诚信政府前10名、中国最具竞争力100强城市、中国最佳投资城市、中国最具海外影响力城市、中国十大最具安全感城市，连续四届获全国社会治安综合治理优秀市和两届“长安杯”城市。

　　五年来，我们重点抓了以下八个方面工作：

　　㈠坚持项目带动，发展后劲进一步增强。五年累计完成投资1778.9亿元。实施市重点项目569个，完成投资772.4亿元，建成项目473个。其中，工业项目244个，完成投资500.3亿元，新钢300万吨薄板、中冶南方冷轧硅钢、赛维硅料硅片、瑞晶太阳能电池、江锂锂盐及电解镍、分电33万千瓦机组等重大产业项目相继建成投产；农业和农业产业化项目65个，完成投资32.1亿元，推进了恩达1万吨微生物脱胶精干麻、润合百万头生猪和万吨肉制品深加工等一批重点项目；交通及城市建设项目181个，完成投资196.3亿元，建成了长青南路立交、科环路、中山路和第四水厂、城东污水处理厂等一批重大基础设施项目，高速公路通车里程达到134公里，洋坊站扩能改造、杭南长客专（新余段）建设扎实推进，新吉铁路列入铁道部建设规划；社会事业及服务业项目79个，完成投资43.7亿元，市体育中心、新余学院、人民医院新院、市一中新校区等投入使用。五年来，报批用地6.4万亩，其中争取省级重大项目用地指标2.4万亩。新探明1亿吨铁矿、2亿吨煤炭资源储量。

　　㈡坚持工业立市，工业经济进一步壮大。着力抓好三大产业发展。做大做强钢铁产业。钢产量达850万吨，是“十五”末的2.1倍；主营业务收入达600亿元，是“十五”末的3.6倍。探索推进新能源产业。已形成3000兆瓦硅片、1.1万吨硅料、1000兆瓦太阳能电池片、200兆瓦组件、200台套螺杆膨胀动力机产能，主营业务收入达330亿元。规划推进新材料产业。主营业务收入达45亿元。着力抓好三大园区建设。全市园区实现工业主营业务收入647亿元，是“十五”末的12倍。新余高新技术产业园区晋升为国家级高新技术产业开发区，2010年实现工业主营业务收入450亿元、工业增加值110亿元、财政总收入10.9亿元，分别是“十五”末的11倍、8.8倍和9.1倍，年均分别增长61.5%、54%和55.5%。分宜工业园实现工业主营业？？收入82亿元、工业增加值21亿元、财政总收入1.8亿元，分别是“十五”末的25倍、26.3倍和25.7倍，年均分别增长91%、92%和92%。新余经济开发区作为省特钢产业基地，实现工业主营业务收入60亿元、工业增加值12.2亿元、财政总收入1.2亿元，分别是“十五”末的6.6倍、6.7倍和3.6倍，年均分别增长46%、46%和29%。着力抓好重点企业培育。规模以上工业企业由245户增加到371户，主营业务收入过亿元企业由25家增加到125家，全市主营业务收入过10亿元的企业9户，过百亿元的2户，纳税过千万元的25户。赛维LDK、新钢股份、赣锋锂业成功上市。

　　㈢坚持以城带乡，城乡统筹进一步推进。被列为全省统筹城乡经济社会发展一体化试点市，荣获2009年度全省新型城镇化先进市第一名。委托中国城乡规划研究总院编制的市域总体规划省政府已审议通过。委托财政部科研所编制的经济发展战略规划得到专家的肯定。对仙来大道、抱石大道、仰天岗大道、仙女湖大道、孔目江湿地公园等进行了新建或改造，加快推进了高新区、孔目江新城、袁河生态新城、仰天岗国际生态城、高铁新区等建设。城市生态环境建设加强。建成区绿地率达50.46%，绿化覆盖率达51.67%，人均公园绿地面积达16.01平方米。仰天岗森林公园被评为国家森林公园，孔目江湿地公园是全省唯一的国家级湿地公园。统筹城乡发展稳步推进。构建了“一个总体方案、九个配套文件”的政策框架，开展了宅基地换房、城乡一体化户籍改革、扩权强镇改革试点，率先在全省基本实现村村通水泥公路、客车、广播电视、程控电话。加强“三农”工作。粮食生产实现连续七年增长，粮食流通平稳有序。农业产业化步伐加快，现代农业科技园晋升为国家级农业科技园。五年新增国家级龙头企业2家、省级龙头企业7家；新增新余蜜桔5万亩、高产油茶7.7万亩、优质苎麻4.7万亩。加强农田水利基础设施建设。五年共改造中低产田12.5万亩，其中建设高标准农田1.8万亩，实施“造地增粮富民工程”增加耕地6.9万亩，完成病险水库除险加固141座、小农水项目建设64个。解决农村27.1万人饮水安全问题。

　　㈣坚持优化结构，第三产业进一步发展。出台系列政策措施，着力营造鼓励、支持第三产业加快发展的良好氛围。大力发展旅游业，仙女湖风景名胜区、仰天岗国家森林公园建设有序推进，启动了“中国洞村”、百丈峰景区、圣集寺、体育公园等重大旅游设施建设，旅游总收入25.5亿元，是“十五”末的2.2倍，年均增长17.3%。大力发展现代金融业。先后引进2家异地银行设立分支机构，各项贷款余额531.8亿元，是“十五”末的5.3倍，年均增长39.6%。大力发展商贸物流业。洪客隆、步步高等大型商业网点建成营业，完成了沙土、环青等15个农贸市场的新建、扩建或改造。一批物流中心投入运营，汽车货运业快速发展，公路货物运力年均增长20%。

　　㈤坚持科技引领，人才建设进一步强化。启动实施了科技创新“543211”工程。着力建设国家硅材料及光伏应用产业化基地、螺杆膨胀动力机高新技术产业化基地。推进国家光伏工程技术研究中心、新钢国家级企业技术中心、国家光伏基础材料及应用产品质量监督检验中心建设；江西开昂新能源研究所落户我市。新钢、赛维获准设立博士后工作站。全国螺杆膨胀发电机标准化技术委员会和国家稀土永磁电机工程技术研究中心力德风电研究所建设积极推进。我市先后与一批科研院所签订了战略合作协议。大力实施人才引进培养“十百千万”工程。举办了“中国工程院院士新余行”活动。聘请了11名院士和13名专家学者担任市政府顾问。我市“人才引领产业，产业聚集人才”的做法得到中央领导的肯定，并在全国人才工作会上作典型发言。

　　㈥坚持改革开放，发展活力进一步彰显。国企改革任务提前完成，职工得到妥善安置，改制企业焕发生机。政府机构改革全面完成；事业单位分类和机构改革不断深化；文化体制改革加快推进；统筹城乡发展等区域性综合配套改革试点全面铺开；集体林权制度改革走在全省前列；财政、金融等改革步伐加快，市农村合作银行增资扩股加快推进。对外开放水平不断提高。新余海关和江西出入境检验检疫局新余办事处高效服务。2010年实际利用外资5.3亿美元，是“十五”末的7.4倍，年均增长49.1%；实际引进内资243.9亿元，是“十五”末的4.8倍，年均增长36.6%。进出口企业由35家增加到136家；外贸出口20.1亿美元，是“十五”末的47.9倍，年均增长116.8%。

　　㈦坚持以人为本，幸福指数进一步提高。五年累计新增城镇就业14.3万人，就业率达96.5%；“招工、培训、就业”三位一体工作模式在全国推广。社会保障体系不断健全。城镇医疗和养老保险参保率、资金征缴率、资金社会化发放率均达100%。在全省率先为80岁以上老人发放高龄补贴；率先实行农民低保制度；率先对村干部和失地农民实行养老保险。大力实施“光明·微笑”工程，累计为4171例白内障患者、179例唇腭裂患者实行免费手术治疗。开展了儿童白血病、先天性心脏病免费救治试点。五年新建廉租住房4273套、经济适用住房32.9万平方米，改造城市棚户区50.8万平方米。广播剧《重返鄱阳湖》获全国“五个一工程”奖，少儿舞蹈《快乐奶仔》获第十五届全国群星奖。圆满承办十二届省运会，市体育中心被命名为国家级全民健身活动中心。食品安全城市创建取得显著成效。安全生产、信访维稳、行政执法和社会治安综合治理等各项工作扎实开展，社会和谐稳定。人口计生工作走在全省前列，被列为全国人口和计划生育综合改革示范市、全国流动人口计划生育基本公共服务均等化试点城市。蒋国珍被列为全国重大宣传典型。

　　㈧坚持效能为先，服务环境进一步改善。在全市开展了“机关效能年”和“创业服务年”活动，取消市级行政审批、行政许可事项105项。乡镇全部建立了便民服务中心和公共资源交易站。市政府及组成部门自觉接受市人大及其常委会的依法监督和市政协的民主监督。五年来共办理人大代表建议296件、政协委员提案1305件。政务公开加快推进，做到了凡与人民群众切身利益密切相关的事项均在网上公示，并公开接受查询。依法行政扎实推进，法治政府建设取得明显成效。“廉政阳光工程”深入推进，反腐倡廉进一步加强。文化体育、广播影视、新闻出版、统计审计、机构编制、外事侨务、民族宗教、涉台事务、气象服务、防空防震、减灾救灾、国防动员、方志档案、决策咨询、社科研究、妇女儿童、老龄、残疾人等各项工作取得了新成绩。

　　各位代表，过去的五年，我们经受了国际金融危机、低温雨雪冰冻灾害、特大洪涝灾害的严重冲击，克服了体制转轨、经济转型中的重重困难，实现了全市改革开放和现代化建设新的目标。成绩的取得来之不易！这是全市人民在省委、省政府和市委的正确领导下开拓创新、团结拼搏的结果，是历届班子真抓实干、务实推进的结果，是人大依法监督、政协民主监督和全力支持、积极建议的结果，是驻市中央省属单位、部队、武警、各民主党派、工商联、无党派和社会各界人士、离退休老同志、广大客商和港澳台同胞、华人华侨齐心协力、共同努力的结果。在此，我代表市人民政府，向所有关心新余发展、投身新余建设的同志们、朋友们表示衷心的感谢和崇高的敬意！

　　回顾过去五年的工作，我们也清醒地认识到，全市经济社会发展还面临不少的困难和问题：经济总量偏小，城市核心竞争力、文化影响力还不强，与赣西区域中心城市的要求还有较大差距；新能源产业发展不足，产品应用示范不够，与国家新能源科技城的要求还有较大差距；人才、物流、用地等要素制约尚未缓解，节能减排和资源环境承载压力还在加大，与转变经济发展方式的要求还有较大差距；社会建设和城市管理面临新的课题，政府工作人员的服务意识、服务水准与加快发展的要求还有一定差距。对此，我们一定要高度重视，采取得力措施，切实加以解决。

　　二、“十二五”规划的主要目标

　　“十二五”时期，是我市积极融入鄱阳湖生态经济区、加快建设国家新能源科技城、努力建成赣西区域中心城市的重要时期，是加快转变经济发展方式，努力实现全面小康和城乡一体化的关键时期，是深化改革开放，推动科学发展、加快发展的战略机遇期。按照市委《关于制定新余市国民经济和社会发展第十二个五年规划的建议》要求，《新余市国民经济和社会发展第十二个五年规划纲要（草案）》提出我市经济社会发展的主要目标：一是积极融入鄱阳湖生态经济区，加快建设国家新能源科技城；二是致力建设赣西区域中心城市；三是在全省率先实现全面小康、率先实现城乡一体化。我市是鄱阳湖生态经济区的重要成员，要紧紧抓住这一重大机遇，加强与《鄱阳湖生态经济区规划》的对接，力争在争取国家支持政策、重大项目、资金投入等方面有重大突破。建设国家新能源科技城，是当前和今后一个时期我市经济发展的主线。我市要通过进一步做大做优做强光伏、动力与储能电池、风电和节能减排设备制造四大新能源经济板块，提升产业规模和竞争实力，加大示范应用力度，努力建成全国新能源领域的技术、制造、信息和人才培养中心。建设赣西区域中心城市，是国家《促进中部地区崛起战略规划》、《鄱阳湖生态经济区规划》对我市的定位。要加快做大区域经济总量，增强城市承载能力、辐射能力，构建赣西区域的经济中心、文化中心、商贸中心与物流中心。在全省率先实现全面小康、率先实现城乡一体化，是省委、省政府对我市的期盼。要全面加强经济、政治、文化、社会和生态文明建设，使经济更加发展、民主更加健全、科教更加进步、文化更加繁荣、社会更加和谐、人民生活更加殷实，在全省率先基本实现全面小康社会；扎实推进统筹城乡经济社会发展一体化试点工作，到2015年，在全省率先基本实现城乡规划布局、经济发展、基础设施、就业社保、公共服务、生态环境、社会管理一体化。

　　围绕上述目标，综合考虑我市发展的趋势和现实条件，《纲要（草案）》提出全市“十二五”经济社会发展主要指标是：到2015年，生产总值达1700亿元，年均增长15%；财政总收入280亿元，年均增长28.3%；固定资产投资1650亿元，年均增长20%；社会消费品零售总额258亿元，年均增长18％；外贸出口总额50亿美元，年均？？长20%；规模以上工业主营业务收入5000亿元，年均增长32.4％；城镇居民人均可支配收入32000元，年均增长13％；农民人均纯收入14100元，年均增长13.5%。

　　三、2011年的工作任务

　　2011年是中国共产党成立90周年，也是“十二五”时期开局之年。全市经济社会发展的总体要求是：以党的十七大、十七届三中、四中、五中全会、中央经济工作会议和省委十二届十四次全会精神为指针，以科学发展为主题，以积极融入鄱阳湖生态经济区、加快建设国家新能源科技城为主线，切实转变经济发展方式，坚持工业立市、科教兴市、人才强市不动摇，坚持重大项目拉动、改革开放促动、城乡统筹联动不动摇，坚持“实现全市人均期望寿命80岁”与各项民生事业发展不动摇，着力推进新型工业城、科技创新城、生态文明城建设，着力建设赣西区域中心城市，着力提升人民群众幸福指数，为顺利实现“十二五”规划目标、加快实现全面小康和城乡一体化打下坚实基础。经济社会发展的主要预期目标是：生产总值750亿元，增长15%。财政总收入105亿元，增长30.2%；地方财政收入63亿元，增长26%。固定资产投资856亿元，增长30%。社会消费品零售总额133.3亿元，增长18%。外贸出口总额25.16亿美元，增长25%。实际利用外资6.37亿美元，增长20%；引进内资317亿元，增长30%。规模以上工业增加值400亿元，增长35%；规模以上工业主营业务收入1800亿元，增长46.3%。城镇居民人均可支配收入19615元，增长13%；农民人均纯收入8486元，增长13.5%。物价水平总体保持稳定。人口自然增长率控制在省定目标以内。力争在省政府组织的“四项考核体系”综合评比中主要指标位居全省前列。努力实现“十个提高”：一是财政收入占GDP的比重由12.8%提高到14%。二是税收占财政收入的比重由84.3%提高到85.3%。三是工业增加值占GDP的比重由57.5%提高到58.3%。四是三大产业主营业务收入占规模以上工业主营业务收入的比重由79.3%提高到82.2%。五是产业项目投资占城镇固定资产投资的比重由51.3%提高到71.4%。六是高新技术产业增加值占GDP的比重由15%提高到16%。七是非公有制经济增加值占GDP的比重由62.2%提高到68%。八是主要农产品深加工比例提高5个百分点。九是城镇化率由58.5%提高到62.5%。十是提高节能减排水平，单位生产总值能耗和二氧化碳排放强度控制在省定目标范围之内；化学需氧量、二氧化硫、氨氮排放量均减少1.5%左右，氮氧化合物排放量减少1%左右。

为实现上述目标，重点做好以下七个方面工作：

　　㈠推进项目建设，增强发展后劲

　　1、实施一批重点项目。计划安排1000万元以上投资项目227个，总投资2132亿元，年度计划投资655亿元。其中市重点项目88个，总投资1181亿元，年度计划投资256亿元，增长28%。重点抓好袁河生态新城、高铁新区、孔目江新城、仰天岗国际生态城、江西省职业教育园区等项目建设。突出抓好十大基础设施项目建设：⑴仙女湖大道西延段、清宜公路货运通道10月前竣工通车。⑵环城南路年底垫层通车。⑶虎踞大道完成土石方、垫层施工。⑷罗坊至水西一级公路改造10月动工。⑸新余大桥年底竣工。⑹罗坊大桥8月底通车。⑺分宜县城引水工程年底竣工。⑻新建市区雨污分流管网。⑼新建赣西中心物流园区。⑽新建500千伏第二输变电工程。

　　2、申报一批重大项目。积极做好项目申报，力争有更多的项目进入全省、全国的项目库。赛维新增1.5万吨硅料、白梅水库、新电2台66万千瓦发电机组项目获得国家立项。

　　3、储备一批优势项目。按照国家产业政策和发展规划，立足新余实际，着力编制一批重大项目，加强策划运作，为项目争取及储备打好基础。

　　4、引进一批重大项目。围绕工业三大产业、现代服务业、现代农业和基础设施建设等重点领域，充分发挥三大园区优势，瞄准沿海发达地区、香港、台湾等重点地区和央企，积极引进更多的大企业、大项目。

　　㈡推进工业立市，集聚优势产业

　　1、抓好三大产业发展。充分发挥龙头企业带动作用，着力延伸产业链。钢铁产业以新钢为龙头，发展高强度船板、冷轧电工钢、压力容器板等，提升产品附加值，全力打造全国系列板管精品基地及华东地区带钢、钢丝生产基地；全年钢产量达1000万吨，实现主营业务收入720亿元，增长24.1%。新能源产业以赛维等为龙头，围绕光伏、动力与储能电池、节能减排设备制造、风电等四大板块，重点完善光伏产业链，形成5000兆瓦多晶硅片、1.8万吨多晶硅料、2000兆瓦太阳能电池片、500兆瓦组件、500台套风力发电机和500台套螺杆膨胀动力机产能；实现主营业务收入700亿元，增长133.3%。新材料产业以江锂为龙头，重点发展电解镍及制品、金属锂及制品、各类复合材料；实现主营业务收入60亿元，增长50%。

　　2、抓好三大园区建设。提升园区承载力，加快项目集聚。新余高新技术产业开发区重点建好光电、动力与储能电池产业等五大“区中园”，争取设立出口加工区；积极推进国家新型工业化产业示范基地建设，高新技术产业增加值占工业增加值比重达到71%，提高2个百分点；实现工业主营业务收入850亿元、工业增加值215亿元、财政总收入28亿元，分别增长88.9%、65.4%和156.3%。分宜工业园要按国家级开发区标准建设，打造新材料特色产业园，实现工业主营业务收入120亿元、工业增加值30亿元、财政总收入2.8亿元，分别增长40%、34%和45%。新余经济开发区以钢铁精深加工为主，申报省级工业园区，打造金属材料高新技术特色产业园，实现工业主营业务收入60亿元、工业增加值12亿元、财政总收入1.8亿元。进一步加大园区路网、水网、电网、通信网等基础设施建设力度，加快推进污水处理、天然气入园项目建设。

　　3、抓好重点企业帮扶。实施“十百千亿”工程。采取超常规措施支持新钢、赛维向千亿元目标迈进。实施突破性举措扶持瑞晶、升阳、吉阳、江锂、新钢板材、新良盛鼎、中冶南方、新华制品、赣锋锂业、亿铂科技等10家企业向“十二五”时期主营业务收入分别过百亿元奋斗。实现钢铁、光伏、动力与储能电池三大产业主营业务收入分别超千亿元。实施特殊政策、特殊服务、特殊办法，引导各类要素向优强企业、优势产品和优秀企业家集中，推动企业裂变式扩张。今年新钢、赛维主营业务收入各突破400亿元，瑞晶实现百亿元。重点抓好新钢1550毫米冷轧工程、赛维1000兆瓦太阳能电池及新增2000兆瓦多晶硅片、瑞晶四期20条电池片生产线、升阳780兆瓦太阳能电池片、瑞昌晟5000兆瓦多晶硅铸锭切片、沣森科技固态硬盘及第四代通信基带芯片生产线、吉阳1000兆瓦太阳能电池片、赣锋锂业1.5万吨氯化锂及5000吨高纯碳酸锂、江镍高纯材料镍带镍丝深加工、亿铂科技打印耗材生产等10个项目。

　　㈢推进城乡一体，加快统筹进程

　　1、健全城乡规划。以城市总体规划为龙头，完善专项规划，严格规划管理，切实发挥规划在城乡建设中的引领作用。完成高新区、仙女湖区、孔目江生态经济区、袁河生态新城、高铁新区分区规划及控规编制，做到控规全覆盖。做好重点区域、重点地段、重要节点的城市设计和修建性详规。进一步抓好乡镇规划体系编制，全面完成11个中心镇总体规划编制，突出抓好罗坊、双林两镇近期建设规划和修建性详规。

　　2、建设低碳城市。加强节能减排，严格执行目标责任制，落实节能减排“一票否决制”，力争成为全国低碳经济试点城市。加快新钢和工业园区污水处理厂建设，淘汰落后产能，全面完成省政府下达的主要污染物减排任务。加强新能源产品开发应用，完成新欣大道太阳能路灯改造，实施15兆瓦光电应用和200万平方米的光（地）热应用项目，重点抓好高新区11.88兆瓦光伏发电集中应用示范和江西自然科学博物馆3兆瓦光电项目建设。巩固国家森林城市、全省生态园林城市创建成果，积极创建全国生态文明城市。深化造林绿化“一大四小”工程效益，确保完成10万亩造林任务，重点建设袁河生态新城。

　　3、完善城乡交通。全面推进乡村公路改造。巩固现有城乡公交发展成果，推进城乡客运一体化。加强地下停车场建设，打通长青路、白竹路等“断头路”，创建国家一级道路畅通城市。启动仙女湖大道东延段改造工程，加快环城西路建设；推进新余至新干赣江码头货运通道建设，力争开工建设新吉铁路。着力构建城市“三环”快速通道，加快构建新余主城区与周边市县“1小时经济圈”和乡镇“半小时经济圈”。

　　4、提升城市品位。按照“改造老城区，建设生态新区，提升高新区”的思路，集中展示城市品位。加快棚户区和城南片区小街小巷改造，美化亮化中山路、北湖路。突出建设好十大标志性建筑：文化中心、高铁新余站、江西自然科学博物馆、迎宾馆、农村合作银行金融大厦、市民中心、城市规划展示馆、创业大厦、新余国际商城、抱石文化创意园。加强城市管理，完善社区建设，深化物业管理，推进“城市管理社区化、社区管理物业化、物业管理市场化”。开展环境秩序“百日综合整治行动”，巩固卫生城市创建成果。加强融资平台建设，有效经营城市土地，盘活城市资产，优化城市资产组合。抓好新增建设用地报批，新增报批1万亩。依法依规节约集约用地，做好土地卫片执法检查和城乡建设用地“增减挂”工作。

　　5、壮大县域经济。支持县区差异竞争、错位发展，引领县区扬长避短，找准比较优势，培育特色经济，走有特色、重创新、快发展之路。分宜县重点推进动力与储能电池、新材料产业，确保生产总值增长16%，规模以上工业主营业务收入增长50%；财政总收入24亿元，增长31.8%。渝水区重点发展钢铁、光伏、机械制造、金属材料产业，确保生产总值增长16%，规模以上工业主营业务收入增长49.1%；财政总收入24亿元，增长31.3%。仙女湖区重点发展文化教育、旅游休闲产业，确保生产总值增长20%，旅游人数增长28%；财政总收入3.3亿元，增长22.2%。孔目江生态经济区重点打造科研创新、生态休闲基地，确保生产总值增长26%；财政总收入5亿元，增长37.9%。

　　6、夯实现代农业。全力打造粮食、果业、蔬菜、苗木四大农业产业化基地。粮食播种面积150万亩、总产量60万吨，确保粮食安全；新增蜜桔1万亩、高产油茶2.8万亩、苗木2万亩、花卉2000亩。市级以上龙头企业达100家，农民专业合作社达350家。发挥国家级农业科技园示范作用，建设以现代畜牧产业园、科农公司、渝洲生态农业公司为龙头的全国一流畜牧产业基地。扎实开展“农田水利基本建设年”活动，力争开工建设白梅水库，抓好15座小（一）型水库除险加固，启动203座小（二）型水库除险加固，继续抓好袁河堤除险加固，完成南安江防洪主体工程、杨桥河城西防洪工程建设，推进渝水区、分宜县小型农田水利重点县建设，抓好中小河流治理，建设高标准农田1.5万亩，完成土地整治9.5万亩。继续安排300个新农村建设点，深入实施“百万树木进村入户”、农村新房型推广和农村清洁工程。建设十大新农村建设示范点，每个县区必须有2-3个先进典型。

　　7、建设示范乡镇。以完善镇村规划和提升功能为核心，以罗坊、双林两个示范中心镇建设为重点，推进11个中心镇建设，加快垃圾污水处理和文化、教育、医疗等基础设施建设，确保两个示范中心镇项目建设总规模突破10亿元，集镇人口增长8%。加强土地管理，启动农村宅基地确权发证工作，搞好农村土地流转，全市土地流转面积达到15万亩。

　　㈣推进三产发展，繁荣城市经济

　　1、大力发展商贸流通业。重点抓好10个商贸网点建设：胜利路步行街、劳动北路商务中心、大润发商贸城、洪城大厦、步步高城北购物中心、新余国际商城、广城家居广场、名嘉广场、赣西万商红国际商贸物流中心、新华书店片区文化商务中心。加快配送中心和农资市场建设，加大“万村千乡市场工程”农家（资）店创建力度，加强乡镇集中配送中心市场规划建设；加快城郊大型农副产品及物流市场建设；发展农超对接企业，推进乡村农贸市场建设改造，做好家电下乡和家电以旧换新工作。

　　2、大力发展现代物流业。加快赣西物流园区、良山钢材物流市场、仰天岗松江钢贸城、穗东物流中心、冷链物流园建设，完成华澄现代物流园一期建设，推进九鼎汽车大市场整体搬迁，加快仙女湖汽车城建设。规范汽车货运业发展，公路货物运力增长10%以上。

　　3、大力发展现代金融业。引进1家异地银行在我市设立分支机构，组建2家村镇银行、1家股权投资基金公司、2家小额贷款公司、2家担保公司，年末贷款余额突破600亿元、企业上市等直接融资余额突破230亿元。推进企业上市，力争建和混凝土、赛维硅料、吉阳新能源年内上市，科农种猪向证监会申报上市材料，恩达家纺进入上市辅导期，引导更多企业做好上市准备。启动城市公众一卡通工程。

　　　4、大力发展文化旅游业。加强以仙女湖风景名胜区为龙头的旅游项目开发及配套设施建设，抓好各区域旅游产品开发，实现旅游层次和水平的大提升。重点抓好圣集寺、体育公园、抱石文化创意园等项目续建，抓好商务、休闲设施建设，开发生态养生城、旅游城、影视城、文化主题公园等一批重大旅游项目。加快仰天岗国家森林公园建设。推进“中国洞村”、袁河生态观光游、孔目江湿地公园三期、百丈峰？？区等旅游项目建设。大力发展工业旅游、城市观光和乡村休闲旅游，建设一批美丽乡村示范点，新开发10个农家乐项目。全市旅游总收入增长29%。

　　㈤推进改革开放，创新体制机制

　　1、推进重点领域改革。稳步推进医药卫生体制改革，在政府举办的社区卫生服务机构和乡镇卫生院全面实施基本药物制度，并实现零差率销售；以市中医院为试点，推进公立医院改革。积极推动文化体制改革，鼓励和引导非公有制经济进入文化市场，发展新型文化业态，满足多样化社会需求。新余日报社、市电视台、电台、歌舞剧院保留事业单位性质，实行企业化管理，推进劳动人事、收入分配、社会保障三项制度改革。全面完成国有农垦企业改革。推进城乡一体化户籍管理制度改革。

　　2、创新招商引资模式。建立健全项目库，将项目编制工作分解落实到各职能部门和县区，细化和延伸适合我市产业发展的项目，引导更多的资金投向优势产业和现代服务业。做好三大产业招商。完善市政府主办、县（区）政府和管委会承办、龙头企业和产业园区协办的招商引资推介会模式，强力引进一批大企业、大项目。重点推进专业招商，实行“划分区域、核定任务、全程服务”的招商模式，以专业招商人员为主体，组建一批产业招商队伍，实行驻点、定向招商，专业招商完成全市招商引资总额的60%以上。突出抓好与央企对接，紧盯世界500强、中国500强、大型跨国公司及行业龙头企业，央企落户项目10个、投资总额30亿元以上。把现有企业增资扩股作为招商引资的重要抓手，支持企业加快发展，做大做强。

　　3、积极扩大外贸总量。壮大外贸发展主体，新增5家自营进出口企业。稳定新钢、信诚等企业出口规模，帮助赛维、瑞晶、升阳等企业扩大出口，重点做好高新企业及机电、纺织等产品的出口参展。优化出口产品结构，争取国家更多支持政策，帮助企业引进先进技术装备，促进企业升级改造，提高出口企业产品竞争力。

　　4、加快非公有制经济发展。落实各项非公有制经济发展的举措，降低和放宽个体私营经济市场准入条件，做到各类资本一视同仁。激活民间资本，鼓励民间资本多元投资。非公有制经济实现税收占全市财政总收入的比重达60%以上，新增就业人员占80%以上。

　　㈥推进科技创新，强化发展支撑

　　以科技创新“543211”工程为引领，充分发挥研发及产业优势，为建设国家新能源科技城构筑发展新平台。

　　1、高标准打造科技创新平台。加快建设国家硅材料及光伏应用产业化基地、螺杆膨胀动力机高新技术产业化基地。抓好江西赛维国家光伏工程技术研究中心、新钢国家企业技术中心、国家光伏基础材料及应用产品质量监督检验中心等国家级研发、技术平台建设。积极申报国家动力与储能电池高新技术产业化基地，组建国家光伏知识产权信息中心，建好2个省级企业工程技术中心、7个省级创新型企业和4个省优势科技创新团队，新增省级创新型企业5家，建成6个市级工程技术中心。

　　2、高效能建设产学研对接平台。深度推进与中国工程院、上海交大、武汉大学、中南大学、南昌大学、天津大学等院校的战略合作。成立专门机构，做好服务顾问？？？作，特别是充分发挥中国工程院院士的智囊作用，使三大产业国内外科研成果第一时间得到孵化。为钢铁、新能源、新材料三个中国工程院院士工作站搞好服务，共同设立1-2个企业与科研院所技术研发中心。建立光伏网站、创办新能源报。

　　3、高起点构建成果转化平台。建立投融资合作机制，通过市场运作广泛吸引金融、社会资本投入；支持科研院所联合设立新能源创业风险投资机构，与著名高校联合设立科技项目孵化风险投资机构，引进专业风险投资机构设立分支机构。加强知识产权保护，积极创建国家知识产权示范城，研究制订企业专利管理、知名商标认定和保护等政策，创立一批知名品牌。

　　4、高质量推进人才资源开发。以我市定为全省人才改革发展实验区为契机，强力推进人才建设“十百千万”工程，坚持人才引进与项目引进相结合、人才引进与重大课题攻关相结合、人才引进与重点产业发展相结合、刚性引进和柔性引进相结合。高标准建设好高层次人才公寓。进一步创新人才政策和机制，建立特殊贡献人才政府津贴制，确保人才引得进、留得住、用得好。

　　5、高水平实施教育优先战略。推进义务教育均衡发展，加快幼儿进园、小学进镇、中学进城步伐。大力支持职业教育发展，在校生达10万人。加快江西省职业教育园区建设，完成市职教中心、冶金职业学院二期、渝州科技职业学院仙女湖科技园建设并投入使用，引导本市优质职业院校优先入驻园区；开工建设实训基地，完成园区主干道建设。将新余学院打造成以工科为主导、文理为基础、新能源专业为特色，全国同类院校一流水平的本科院校。

　　㈦推进民生改善，提高幸福指数

　　着力解决事关群众切身利益的问题，努力提高城乡居民的生活质量和收入水平，全面完成省政府下达的民生指标，不断增强人民群众幸福感。

　　1、办好十件实事：⑴新建市十五中和渝水五小、六小，实现所有乡镇有中心幼儿园。⑵推进“村村通自来水”工程。⑶改造城市棚户区（城中村）60万平方米，新建廉租住房12万平方米、经济适用住房120套、公共租赁住房224套，完成租赁补贴4173户。⑷建设万亩无公害蔬菜基地。⑸新建10个城区小公园。⑹启动廖家江、贯早江环境治理工程。⑺加快推进天然气入户工程。⑻工人文化宫投入使用。⑼解决城市社区工作用房。⑽星辉农产品批发市场一期建成并投入使用。

　　2、完善就业保障。健全劳动就业服务体系，实现招工与培训、培训与就业的有效对接，城乡新成长劳动力、就业困难群体与用人单位和公益性岗位的有效对接，创业人员、创业项目与创业孵化基地的有效对接，高校与用人单位、毕业生与用人单位的有效对接。全年新增城镇就业2.21万人，“4050”人员就业0.16万人，新增转移农村劳动力1.35万人，发放小额贷款1.3亿元。

　　3、深化社会保障。全面推进企业社会保险，实现园区企业职工社会保险全覆盖。妥善解决未参保城镇小集体企业职工参加城镇职工基本养老保险问题。进一步降低私营企业和灵活就业人员社会保险参保门槛，破解非公有制经济从业人员参保难题。逐步将养老保险覆盖到社会各个群体，使城镇基本养老保险参保缴费人数达17.05万人，征缴？？额达6.73亿元。加快失地农民基本保障制度建设，落实失地农民的征地补偿、社会保险和就业政策。稳步推进新农保试点，使参保人数达31万人。提高城乡低保对象、农村五保对象保障标准及孤儿养育标准。

　　4、加强健康保障。巩固完善新农合制度，参合率稳定在92%以上，筹资标准每人每年提高到150元，最高支付限额提高到农民人均纯收入的8倍左右。统筹基金使用率和一次性报账率分别达85％、90％以上。推进县、乡、村和城市社区医疗卫生机构标准化建设，提高基层卫生综合服务能力；推进基本公共卫生服务均等化，提高公共卫生服务能力与水平。加强食品药品检验检测能力建设，创建国家级食品药品安全示范城。推行城镇职工基本医疗保险、城镇居民医疗保险、新农合和大病医疗救助“三网一线”管理。引领全民健身运动，大力支持竞技体育事业，办好第七届全国城市运动会相关赛事。

　　5、创新社会管理。健全城乡基层管理和服务体系，提高城乡社区自治和服务功能。完善人民调解、行政调解、司法调解联动的工作体系，建立调处化解矛盾纠纷综合平台。高度重视群众来信来访，畅通和规范群众诉求表达、利益协调、权益保障渠道，最大限度消除不和谐因素。按照“整合资源、信息共享、综合利用”的原则，将数字城管与城市报警监控、交通管理、消防、环保、卫生、安监等政府投资资源进行整合，建设综合应急指挥平台，构建数字城市管理体系，提升城市应急管理水平。巩固国家安全人民防线，加强社会治安综合治理，提高执法队伍素质，支持司法机关公正廉洁执法，不断提高办案质量和效率，依法严厉打击刑事犯罪。加强消防和安全生产，落实企业主体责任，健全事故灾难、公共卫生、食品安全、社会安全事件预防预警和应急处置体系，坚决杜绝重特大事故发生。

　　6、发展社会事业。大力推进文明城市创建活动，成功创建省级文明城市，加快创建全国文明城市。重视科学普及工作，提高全民科学素质。推进全国人口计划生育综合改革示范市建设，抓好流动人口计划生育基本公共服务均等化试点。认真制定实施“六五”普法规划和“十二五”依法治市规划。推进城区有线电视数字化整体转换。高度重视老龄工作，建设居家养老、生态养生示范区。重视支持妇儿事业发展，加强青少年活动场地建设，保障妇女和未成年人的合法权益。关心和支持残疾人事业发展，建立健全保障服务体系。大力做好“双拥”和优抚安置工作，争创全国双拥模范城。

　　四、加强政府自身建设，优化发展环境

　　大力弘扬“开放、创新、争优”的新余精神，深入开展“积极融入鄱阳湖生态经济区，加快建设国家新能源科技城”、“千名干部下基层，排忧解难促和谐”和“发展提升年”主题活动，全面加强政府自身建设。

　　1、提升行政效能。创新管理方式，提升服务水平，不断提高工作效能。全面落实岗位责任制、服务承诺制、责任追究制，完善“一站式办理”、“一条龙服务”、一次性告知、限时办结制。推进行政审批制度改革，进一步精简和下放审批事项。加强绩效管理，完善政府绩效评估指标体系和评价机制。树立“公务员就是服务员”的理念，倡导“五天工作、七天服务”的作风，努力为国家新能源科技城建设服务、为基层发展服务、为人民群？？？服务，对重大项目、重点企业以及涉及企业、群众切身利益的事项，实行“绿色通道”服务，打造全省乃至全国一流的发展环境。

　　2、提高执法水平。把依法行政贯穿到政府工作的各个领域、各个环节，严格按照法定程序、法定权限和法定职责行使人民赋予的权力。坚持合法和高效相统一，不断提高依法行政水平。强化行政执法监督检查，完善行政执法责任制、行政复议制度和评议考核制。完善政府重大事项社会听证公示和专家咨询制度，努力提高决策的预见性、科学性和有效性。深入推进政务公开、政府信息公开和公共企事业单位办事公开，全面推行行政权力网上运行，完善新闻发布制度，保障人民群众的知情权、参与权、表达权和监督权，做到问政于民、问需于民、问计于民，努力使政府的决策得到群众的支持与理解。依法接受人民代表大会及其常委会的法律监督和工作监督，主动接受人民政协的民主监督，及时办理人大议案、代表建议和政协提案，广泛听取各民主党派、工商联、无党派人士和人民团体的意见，高度重视新闻舆论和社会公众监督。

　　3、落实廉政措施。坚持标本兼治、综合治理、惩防并举、注重预防的方针，建立健全惩治和预防腐败体系的各项体制机制。全面落实党风廉政建设责任制，加强反腐倡廉宣传教育，推进风险岗位廉能管理，严肃查处违法违纪案件，坚决纠正损害群众利益的不正之风，预防和遏制腐败现象发生。加强建设工程招投标、财政转移支付、土地和矿产资源使用、政府采购、国有资产转让等公共资源管理权力的监管。强化审计监督、财政监督和行政监察，完善政府采购、招投标与重大项目绩效评估制度。厉行节约、反对浪费，从严控制公务用车、会议、公务接待及党政机关出国（境）经费。各级政府领导要认真落实廉洁自律各项规定，按规定报告个人有关事项，自觉接受监督，严格管好自己的亲属和身边工作人员，一切依法依规办事，做廉洁自律的表率。

　　各位代表！回顾过去，成绩令人鼓舞；展望未来，前景催人奋进！让我们紧密团结在以胡锦涛同志为总书记的党中央周围，在省委、省政府和市委正确领导下，与全市人民一道奋力拼搏，扎实苦干，全面完成今年的各项任务，为实现科学发展、进位赶超、绿色崛起，加快建设国家新能源科技城、打造赣西区域中心城市，在全省率先实现全面小康和城乡一体化而努力奋斗！
